# Microfluidic preparation of optical sensors for biomedical applications

**DOI:** 10.1002/SMMD.20220027

**Published:** 2023-02-12

**Authors:** Qiao Wang, Chong Wang, Xinyuan Yang, Jiali Wang, Zhuohao Zhang, Luoran Shang

**Affiliations:** ^1^ Shanghai Xuhui Central Hospital Zhongshan‐Xuhui Hospital, and the Shanghai Key Laboratory of Medical Epigenetics the International Co‐laboratory of Medical Epigenetics and Metabolism (Ministry of Science and Technology) Institutes of Biomedical Sciences Fudan University Shanghai China

**Keywords:** disease diagnosis, drug evaluation, microfluidics, optical biosensor, organ‐on‐a‐chip

## Abstract

Optical biosensors are platforms that translate biological information into detectable optical signals, and have extensive applications in various fields due to their characteristics of high sensitivity, high specificity, dynamic sensing, etc. The development of optical sensing materials is an important part of optical sensors. In this review, we emphasize the role of microfluidic technology in the preparation of optical sensing materials and the application of the derived optical sensors in the biomedical field. We first present some common optical sensing mechanisms and the functional responsive materials involved. Then, we describe the preparation of these sensing materials by microfluidics. Afterward, we enumerate the biomedical applications of these optical materials as biosensors in disease diagnosis, drug evaluation, and organ‐on‐a‐chip. Finally, we discuss the challenges and prospects in this field.

## Graphical abstract

Diagram of the general idea of the article. It includes mechanism of optical sensors and the involved functional materials, preparation of optical sensing materials using microfluidic and the biomedical applications of these optical materials as biosensors in disease diagnosis, drug evaluation, and organ‐on‐a‐chip.

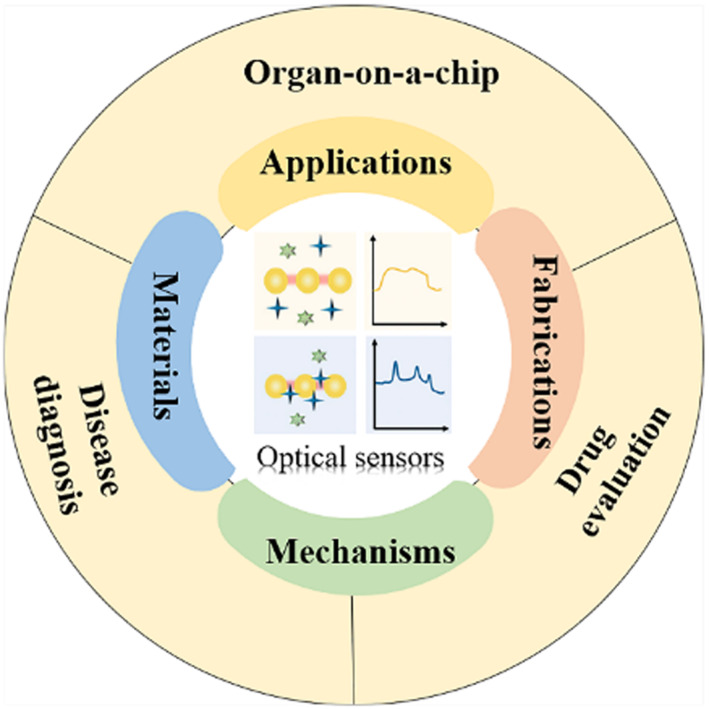

1

**Key points**

- Optical sensors have great applications in many fields.
- The development of optical sensing materials is an important part of optical sensors.
- The microfluidic technique can be used to fabricate optical sensing materials with finely tunable size and structure, by which the materials show excellent optical performance and multiple functionalities.
- Microfluidic‐derived optical sensing materials have great application values in biomedical fields such as disease diagnosis, drug evaluation, and organ‐on‐a‐chip.

## 2 INTRODUCTION

Biosensors are functional materials or devices that could respond to biological activities and convert them into detectable signals.^1^ According to the form of signal conversion and detection, biosensors can be divided into electrochemical sensors, electrical sensors, optical sensors, gravimetric sensors, etc.^2^ Among them, optical biosensors have attracted increasing research interest in recent years because of their high sensitivity, high specificity, dynamic sensing ability, etc. Optical biosensors measure changes of optical associated parameters, such as light wavelength and intensity, when exposed to biological analytes. The optical responses can be based on various mechanisms, including fluorescence, chemiluminescence, surface plasmon resonance (SPR), surface‐enhanced Raman scattering (SERS), to list a few.^3^ Alternatively, structural color is a bioinspired phenomenon that originated from the interactions between light and the micro/nano structure features of an object. Structural color possesses unique advantages such as visually perceptible signal readouts as well as color tunability. More prominently, structural color can be free from photobleaching or quenching, thus providing stable signal readouts.^4^

The sensing process is generally realized by harnessing functional materials with tunable optical readouts.^5^ Nanoparticles (NPs) and their assemblies have made great contributions to the development of optical sensors, including metal NPs in SERS, colloidal crystals for structural color sensing, etc.^6^ The fabrication of these materials is an important aspect, since an appropriate preparation strategy not only enables optical sensor materials to maximize their optical properties but also facilitates the design of their structures and thus extending their functionalities. In particular, to better exploit the optical properties of these materials, microfluidics has been playing an important role.^7^ Microfluidics is an emerging technology that integrates multiple micro‐scale fluid channels in a system and allows for precise control over fluidic behaviors.^8^ Benefited from this capability, microfluidic technology could be employed for the synthesis of nanomaterials with finely controlled size and physicochemical properties, thus achieving better optical sensing performances.^9^ Besides, the diversified fluid configurations could serve as templates for the synthesis of nanocomposite materials or nanomaterial assemblies with higher‐order structures and versatile optical functions.^10^

Due to the rapid development of optical sensors, there are many reviews covering this topic, but few of them emphasize the role of microfluidics in the fabrication of optical sensor materials. Here, we provided a review of microfluidic preparation of optical sensors and their applications in biomedical fields. First, we briefly introduced the commonly adopted optical sensing mechanisms and the functional nano/micromaterials that contribute to such sensing responses. Then, we shifted to the basic concept of microfluidic technology, followed by a systematic description of microfluidic‐based synthesis of sensor materials. Next, we presented the latest progress of the applications of these optical materials as biosensors, including in drug evaluation, disease diagnosis, organs‐on‐chips, etc. Finally, we analyzed the remaining challenges and future development prospects of this field. We believe this review would help foster the development of optical biosensors and inspire the research of microfluidics in fabricating functional biomaterials.

## 3 OPTICAL SENSING MECHANISMS AND SENSOR MATERIALS

Optical sensors have attracted increasing interest and developed rapidly. According to their sensing mechanisms, optical sensors can be categorized into SPR‐based sensors, SERS‐based biosensors, fluorescence‐based sensors, structural color sensors, etc.^11^ In this segment, we explain in detail these types of optical biosensors in terms of their operating principles, characteristics, and the relevant sensor materials.

### 3.1 SPR‐based sensors

**FIGURE 1:**
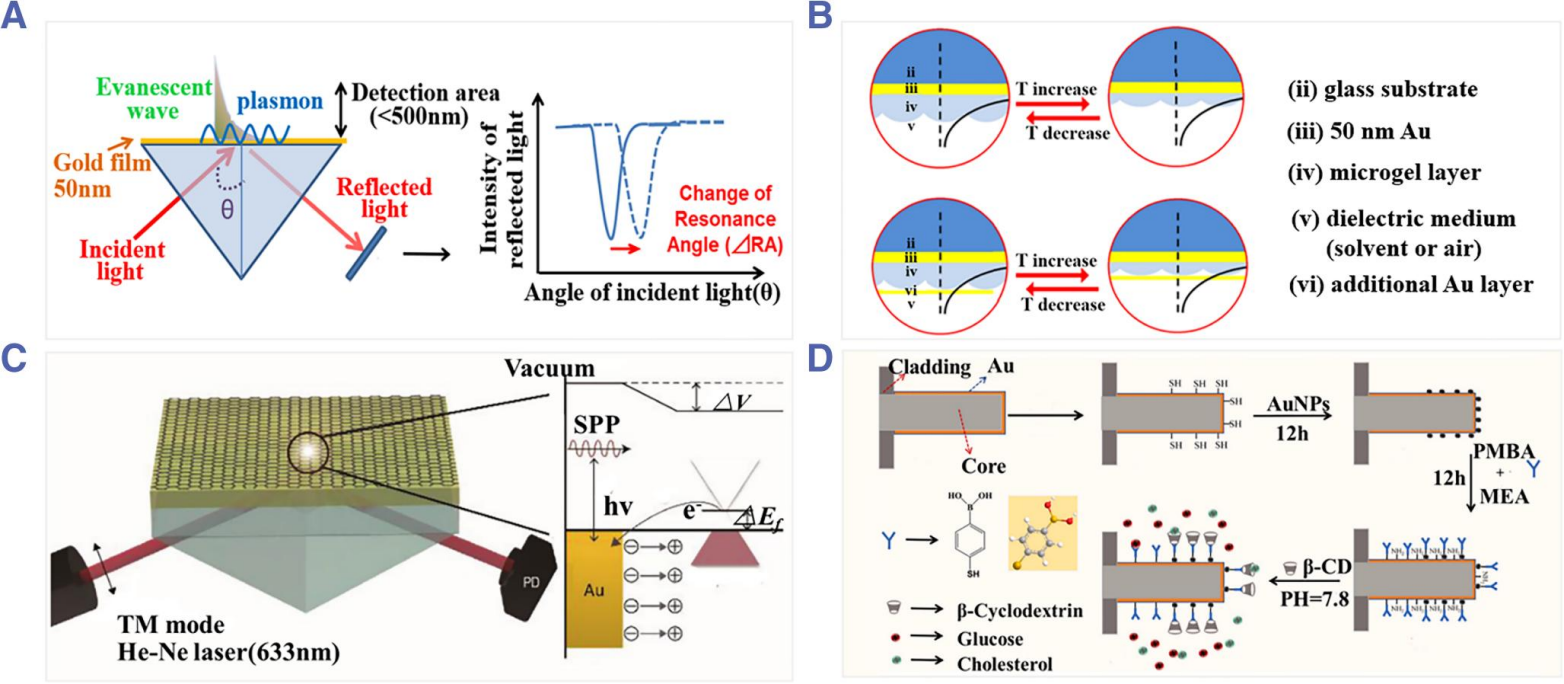
(A) Schematic diagram of the mechanism of SPR. Reproduced under terms of the CC‐BY license.^12^ Copyright 2014, The Authors, published by MDPI. (B) A smart polymer film/Au‐based SPR sensor: (top) cross‐section of the sensor surface coated with a layer of pNIPAm‐based microgel that undergoes temperature‐responsive volume change; (bottom) cross‐section of the sensor surface with an additional layer of Au coated on the pNIPAm‐based microgel providing plasmon coupling. Reproduced with permission.^17^ Copyright 2021, American Chemical Society. (C) Schematic of the graphene‐deposited gold film for enhancing SPR sensitivity. Reproduced with permission.^18^ Copyright 2018, John Wiley and Sons. (D) Schematic diagram of the two‐parameter SPR biosensor and the working principle. Reproduced with permission.^19^ Copyright 2022, Elsevier.

SPR is a well‐studied optical phenomenon that generally occurs at a metal and dielectric interface. Basically, SPR happens when the surface plasmon wave phase‐matches with the evanescent wave. As a result, the intensity of the reflected light dramatically decreases (Figure 1A).^12^ When the wavelength of the incident light is fixed, an angle could be identified corresponding to the minimum of light reflected, which is named the resonance angle.^5^ ^b,^ ^13^ Since the resonance angle is highly sensitive to the refractive index, factors that cause changes in the refractive index could be detected by measuring the displacement of the resonance angle.^14^ Based on this, SPR could be implemented in the detection of molecular binding events, including affinity and kinetics. SPR sensors have the advantages of label‐free and real‐time detection, high sensitivity and selectivity, fast measurement, etc.^15^ Benefiting from the above characteristics, SPR‐based sensors have been widely used for the study of biomolecular interactions and have found applications in biological assays and medical diagnosis.^16^

Au is commonly used as the active metal for SPR‐based sensors.^20^ However, a bare Au thin layer structure suffers from low sensitivity due to insufficient biomolecule absorption, especially for low‐concentration, small molecules. To overcome this, a common practice is to coat or combine other materials into the Au surface to increase the sensitivity. For instance, Serpe and colleagues coated a layer of thermal‐sensitive poly (N‐isopropylacrylamide‐co‐acrylic acid) [p(NIPAm‐co‐AAc)] microgel on an Au film. Besides, another Au layer was deposited on top of the microgel for plasmon coupling (Figure 1B). As a result, the sensitivity of SPR was significantly improved.^17^ Chung et al. prepared graphene‐deposited gold films and elucidated that the presence of graphene could induce electron transfer to the gold film, which contributed to the increase in SPR sensitivity (Figure 1C).^18^ In addition to graphene, other two‐dimensional (2D) materials, such as graphene oxide, MoS_2_, MXene, etc., have been exploited,^20^ ^b,^ ^21^ and their own characteristics as well as contributions to SPR sensing have been well‐studied. Apart from the above methods, SPR performance could also be improved by using gold nanoparticles (AuNPs).^21^ ^a^ Zhao and colleagues presented a dual‐parameter SPR biosensor that relied on Au and AuNPs to excite double resonance. P‐mercaptohenyl boronic caid (PMBA) and β‐cyclodextrin (β‐CD) molecules were further coated through covalent bonding, whose interaction with glucose and cholesterol could cause red‐shift of the respective resonant valley (Figure 1D).^19^

### 3.2 SERS‐based sensors

Raman spectroscopy is a technique that enables identification of the “fingerprints” of molecules, including their chemical composition, conformation, and interactions.^22^ SERS is a phenomenon involving significantly improved Raman signals due to electromagnetic enhancement and chemical enhancement mechanisms. Based on the advantages of ultra‐trace detection, narrow band, low water interference, high stability, and non‐destructive characterization, SERS is widely used in biosensing.^23^

**FIGURE 2:**
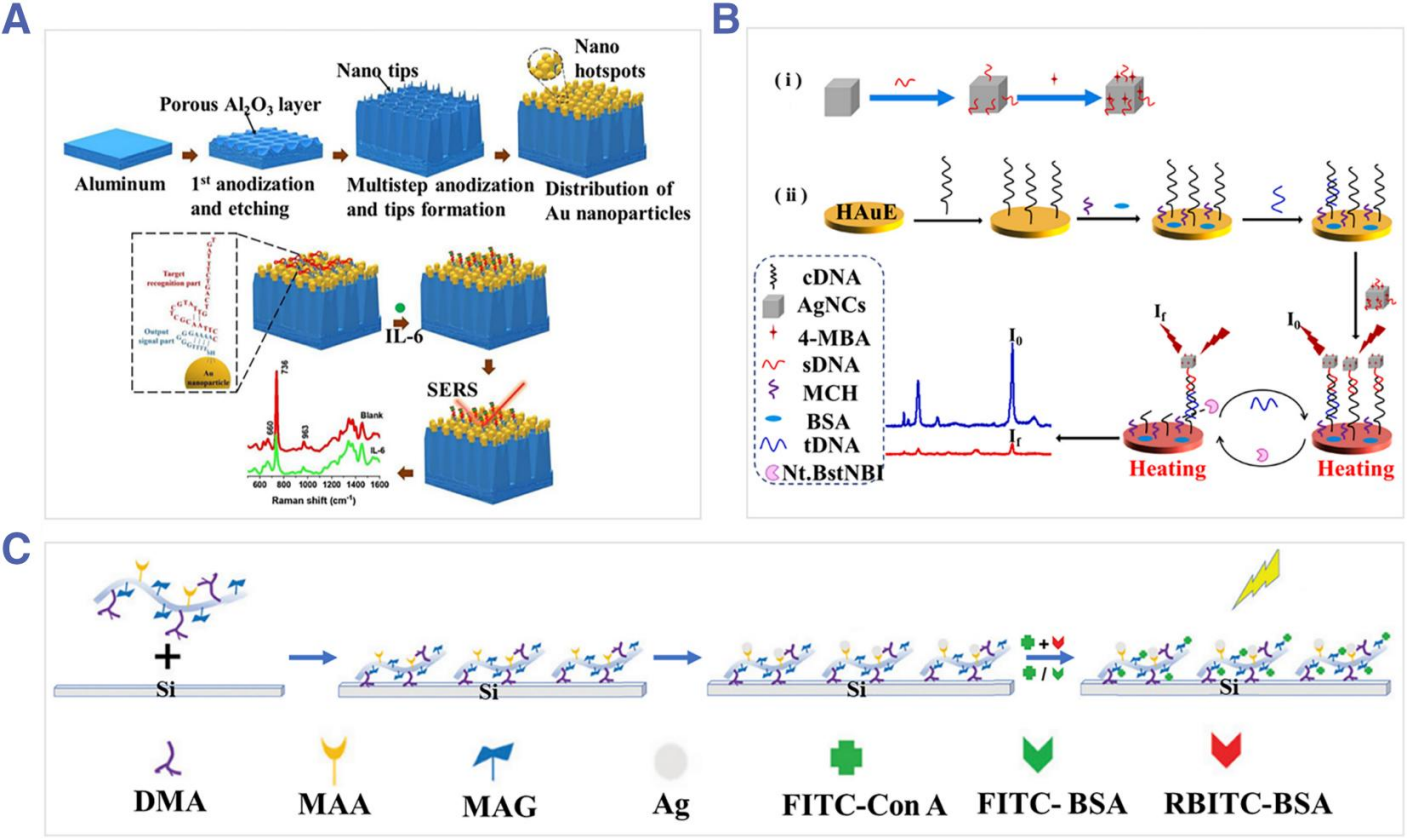
(A) Schematic of the preparation of the AuNP arrays and modification of aptamers for the detection of IL‐6. Reproduced with permission.^27^ Copyright 2021, Elsevier. (B) Schematic of the AgNCs‐based SERS biosensor combined with signal amplification and electrically heated electrode for the detection of DNA. Reproduced with permission.^29^ Copyright 2021, Elsevier. (C) Construction of the polymer‐AgNPs composite SERS substrate. Reproduced with permission.^31^ Copyright 2022, The Royal Society of Chemistry.

For electromagnetic enhancement, metals are dominant SERS substrates because of their high SERS enhancement factor, which enables ultra‐sensitive molecular detection.^24^ Nanostructured noble metal (such as Au, Ag, and Cu) substrates have been extensively studied.^25^ Nanostructures of low symmetry or with sharp geometric features are known to generate “hotspots” effect for SERS enhancement.^26^ For example, Huang's team prepared a highly ordered AuNPs array as the SERS substrate. DNA aptamers were further functionalized for the recognition of interleukin 6 (IL‐6) (Figure 2A).^27^ The dimension and configuration of the nanostructure of the metallic substrate are important factors that affect the SERS efficiency.^23^ ^a,^ ^28^ Besides, the geometric characteristics can also be coupled with auxiliary systems to further improve the performance of SERS sensors. Wu's team built a SERS platform for the detection of DNA by exploiting the hotspots effect of silver nanocubes (AgNCs) and combining it with nicking endonuclease signal amplification as well as the electrically heated electrode technique to achieve high detection sensitivity (Figure 2B).^29^ Moreover, other materials, such as polymers, mesoporous silica, magnetic NPs, etc. could be integrated with nanostructured metals and exert specific functions contributing to SERS detection.^30^ For instance, Chen et al. synthesized glycopolymers via RAFT polymerization, on which Ag nanoparticles (AgNPs) were generated in situ to form a composite SERS substrate. This platform demonstrated specific adsorption of target proteins and selective Raman enhancement ability (Figure 2C).^31^ Apart from metal‐based substrates, some 2D materials have also been explored as SERS substrates based on the chemical enhancement mechanism.^32^

### 3.3 Fluorescence‐based sensors

Fluorescence‐based biosensors are widely used for analyte detection because of their simple design, easy operation, high specificity, etc.^33^ Fluorescence biosensors rely on the use of fluorescent probes that convert biorecognition events into detectable fluorescence signals. The fluorescence sensing mechanisms mainly includes different photophysical processes involving fluorescence resonance energy transfer (FRET), photo‐induced electron transfer, ligand‐to‐metal charge transfer, intramolecular charge transfer, aggregation‐induced emission, etc.^34^ Notably, some nanomaterials possess unique optical properties, such as quantum dots/carbon dots, upconversion nanoparticles (UCNPs), metal‐organic frameworks (MOFs), etc. These nanomaterials bring new opportunities to fluorescence biosensing.

**FIGURE 3:**
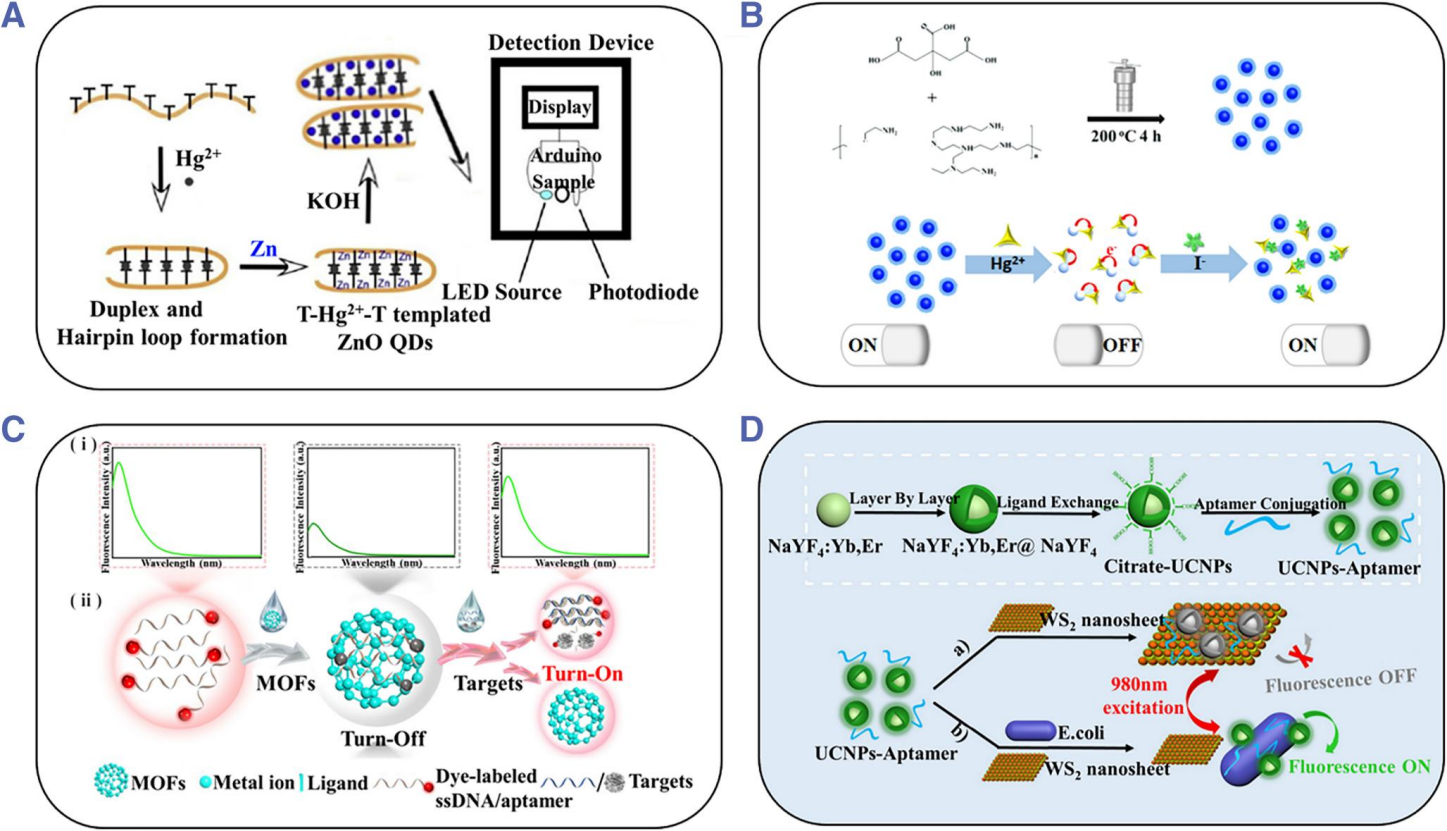
(A) Schematic of the QDs‐based sensing mechanism for the detection of Hg^2+^. Reproduced with permission.^36^ Copyright 2019, Elsevier. (B) Synthesis of the carbon dots‐based fluorescent sensor and the working principle for ion detection. Reproduced with permission.^39^ Copyright 2022, Elsevier. (C) Schematic diagram of MOF‐based switch fluorescence sensing mechanism. Reproduced with permission.^41^ Copyright 2021, The Authors, published by Springer Nature. (D) Schematic showing the conjugation between aptamers and UCNPs and the detection of *Escherichia coli* based on FRET. WS_2_ nanosheet acted as the energy receptor. Reproduced with permission.^42^ Copyright 2020, Elsevier.

Quantum dots (QDs) are zero‐dimensional (0D) semiconductor nanomaterials with unique properties originating from quantum confinement effects, such as large emission quantum yields, size‐controllable emission, and narrow emission bands. Besides, QDs can be surface‐modified to become water soluble.^35^ These characteristics make QDs applicable to optical sensing, and there are significant advances emerging. For instance, Daniel et al. presented a fluorescence sensor based on ZnO QDs for the detection of Hg^2+^. An aptamer was used for the generation of ZnO QDs in the presence of Hg^2+^, precursor, and the reducing agent. The fluorescence enhanced with the increase of Hg^2+^ concentration, thus enabling synthesis and sensing simultaneously (Figure 3A).^36^ Carbon dots (CDs) are 0D carbon nanomaterials typically below 10 nm in size, and have the advantages of outstanding optical properties, excellent water solubility, low toxicity, good bio‐compatibility, etc.^37^ Owning to these properties, carbon dots and their derivatives have important applications in optical sensing.^38^ Zhou and colleagues developed an N‐doped carbon dot‐derived fluorescent sensor for efficient and sensitive ion detection in water. The sensor worked in an “on‐off” manner to detect Hg^2+^ due to electron transfer between Hg^2+^ and CDs, and in an “off‐on” manner to detect I^−^ due to preferential binding between Hg^2+^ and I^−^ (Figure 3B).^39^ In addition, fluorescent silicon NPs, graphene quantum dots, and other materials have also been studied as fluorescent sensors,^40^ which are not elaborated here.

MOFs are composed of metal ions/clusters and organic ligands formed through coordination bonds. MOFs have been attracting much attention due to their ultra‐high porosity, large specific surface area, and structural diversity.^43^ Due to their unique physical and chemical properties, MOFs have great value in gas storage/separation, catalysis, and drug encapsulation/release applications.^44^ In addition, MOFs are promising fluorescent quenching agents that can bind to biological recognition molecules and affect the switching states of fluorescence, as shown in Figure 3C.^41^ Based on this, MOFs have been employed for sensitive and efficient quantitative analysis of various samples.^45^

Upconversion nanomaterials are materials with anti‐Stokes shift properties that can emit short‐wavelength light (visible or ultraviolet) under the irradiation of long‐wavelength light (near‐infrared).^46^ Upconversion nanomaterials have good optical and chemical stability as well as low background interference.^47^ Since upconversion nanomaterials have a strong emission in the short wavelength region and some materials have strong absorption in that region, it is possible to construct fluorescent sensors based on the FRET mechanism.^48^ For example, Chen's team developed a fluorescent sensor for the highly sensitive detection of *Escherichia coli* (*E. coli*) utilizing the fluorescence quenching ability of WS_2_ nanosheets and the emission energy of UCNPs (Figure 3D). This provided a platform for quantitative analysis of *E. coli* in medical diagnostics as well as food and environmental analysis.^42^

### 3.4 Structural color‐based sensors

**FIGURE 4:**
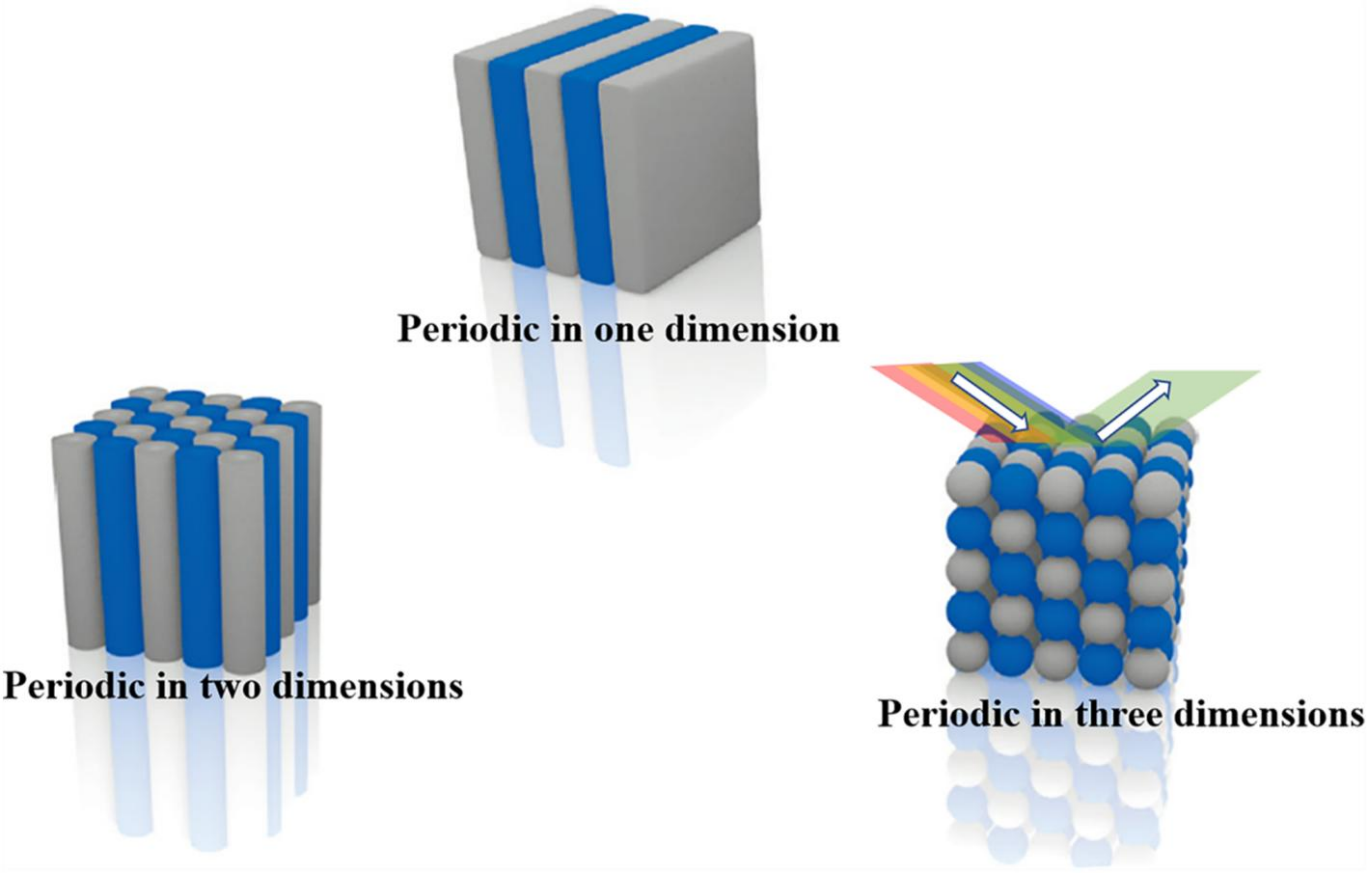
Schematic diagram of the PhCs' periodic structure in one, two, and three dimensions and the generation of structural color. Reproduced with permission.^52^ Copyright 2014, John Wiley and Sons.

Structural color is a phenomenon involving materials with special periodic nanostructures interacting with incident light. Compared with traditional luminescent substances, such as dyes and pigments, structural color materials are not prone to fade, and can avoid quenching, thus being more stable in nature.^49^ These features make structurally colored materials feasible for sensing, decoration, anti‐counterfeiting, etc.^49^, ^50^ Structural coloration includes different mechanisms such as film interference, photonic crystal (PhC), scattering, etc.^51^ PhCs are particularly interesting; they produce visible colors by forbidding the propagation of light at specific wavelengths located in the photonic bandgap (PBG) (Figure 4).^52^ PhC microparticles can be prepared through the confined assembly of colloidal NPs, and are often used in suspension arrays for molecular detection. For example, Shang et al. fabricated PhC microbubbles as encoded microcarriers, which had tunable refraction peak positions. The surface of the barcodes was modified with antibodies to detect the corresponding target molecules, and the results were analyzed by identifying the structural color of the barcodes.^53^ Zhao and colleagues constructed a core‐shell PhC barcode based on GelMA hydrogel. Different cells were cultured on barcodes of different colors and formed spheroids, and this platform was used for drug screening.^54^

### 3.5 Others

In addition to the above‐mentioned sensors, chemiluminescent (CL)‐based sensors, optical resonator‐based sensors, optical fibers‐based sensors, etc. are also of great interest, and each of them possesses its own characteristics. CL‐based sensors can avoid the noise interference resulting from light scattering, thus offering high detection sensitivity.^55^ The working principle of an optical resonator‐based sensor is based on the change of the resonant spectrum in response to the analytes, which is capable of high‐sensitivity, label‐free, and real‐time detection.^56^ Optical fibers have good optical properties due to their unique microstructures and have important applications in sensing.^57^ It is also worth noting that in addition to commonly used sensor materials, new composite materials are being developed,^58^ which continues to promote the development of optical biosensors.

## 4 MICROFLUIDIC FABRICATION OF OPTICAL SENSING MATERIALS

Fabrication of optical materials is fundamental for their applications as sensors. The optical properties of materials largely depend on their physico‐chemical characteristics, such as shape, composition, size, etc. For instance, the optical features of quantum dots strongly rely on their size.^59^ Thus, the synthetic methods of optical materials play an important role. There are many methods for the fabrication of optical materials, among which the microfluidic technology possesses multiple advantages. Microfluidics is an emerging technology that integrates multiple micro‐scale channels in a system enables precise control of fluidic behaviors.^60^ The intricate design of microchannels, the efficient mass and heat transfer of fluids, the dominance of viscous force over inertial forces, the real‐time monitoring capability, and the remarkable surface effects^8^ give it an important position in the fabrication of optical materials.^9^ ^a^ In this section, we introduce microfluidic preparation of optical nano/materials, including direct synthesis in channels as well as fluidic‐templated synthesis.

### 4.1 Direct synthesis of optical nanomaterials in channels

The performance of nanomaterials depends on their physical properties (shape, size, etc.). The controlled fluid mixing in microfluidic channels through either continuous flow or droplet and segmented flow could facilitate the synthesis of nanomaterials with well‐defined properties.^9^ ^a^ In general, there are different models describing the formation mechanisms of NPs, in which microfluidics can control each step involved and thus regulate particle size, size distribution, and surface morphology. In addition, microfluidics can achieve high reproducibility from batch to batch.^61^ Therefore, it plays an important role in the synthesis of optical materials. Metal NPs are commonly used in SPR sensors and SERS sensors, and their composition, shape, and size significantly affect their optical sensing performances.^62^ The most common method for the synthesis of metal NPs using microfluidics is the chemical reduction method, in which a reducing agent and a metal salt solution are injected into a microfluidic chip to fully react.^63^ During the whole reaction process, the reaction conditions, including the temperature, flow rates, and other parameters affect the morphology and size of the final product,^64^ so the design of the microfluidic chip occupies an important position. A commonly used chip design is the serpentine channel, which can enhance the mixing between reagents. Besides, segmented flow‐ or droplet‐based microfluidic systems have been adopted for controllable synthesis of metal NPs with narrow size distribution.

**FIGURE 5:**
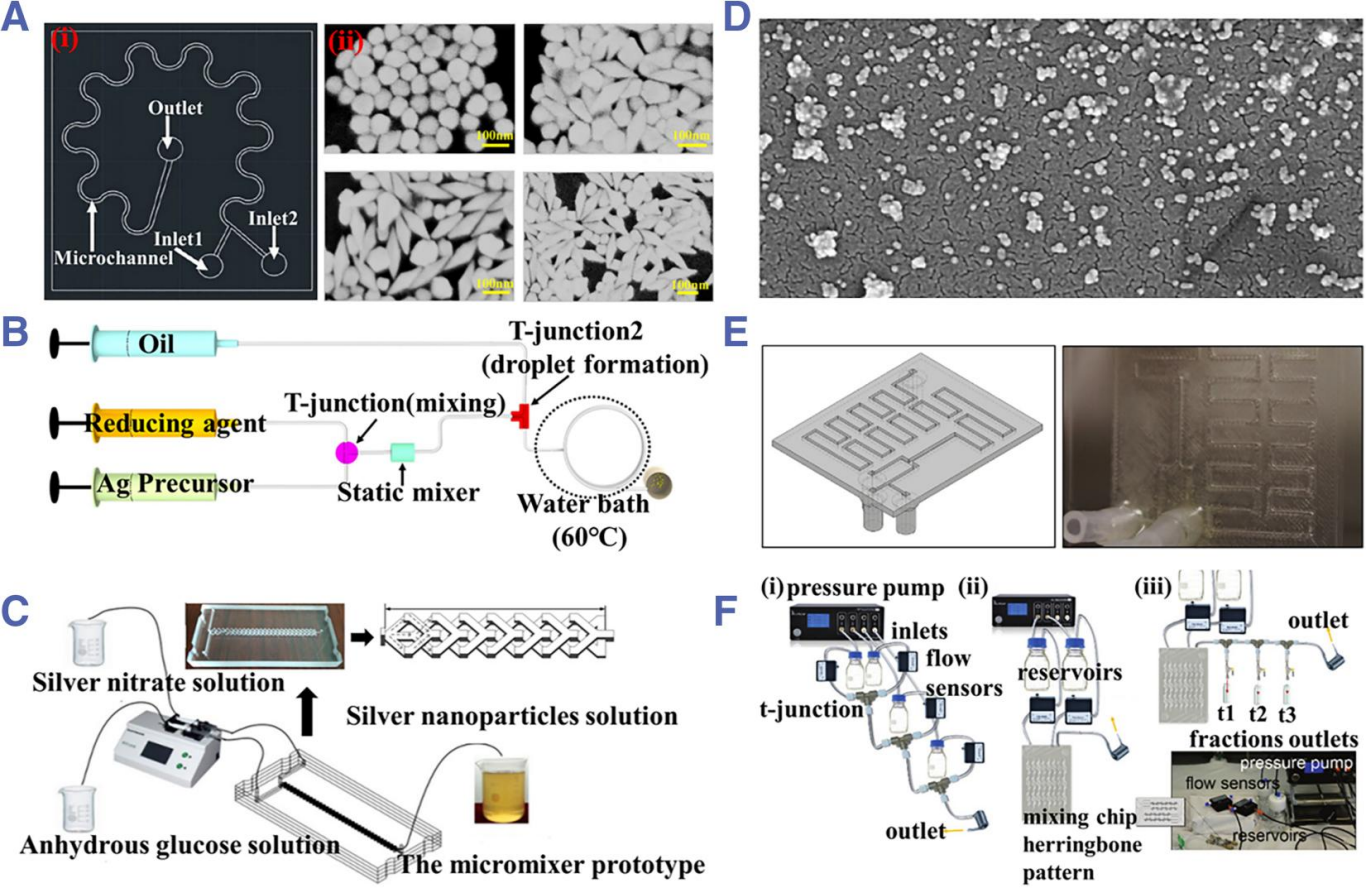
(A) (i) Image of the serpentine microfluidic platform for synthesizing metal NPs. Reproduced with permission.^65^ Copyright 2021, Elsevier. (ii) SEM images of the AuNPs generated with different flow rates. Reproduced with permission.^66^ Copyright 2020, Springer Nature. (B) Tailored microfluidic reactor with fully incorporated T‐junctions, a static mixer, and a heating zone. Reproduced with permission.^67^ Copyright 2018, Elsevier. (C) Schematic diagram of the controlled synthesis of AgNPs by a double‐layer Y‐type micro‐mixer capable of fluid spitting and recombination. Reproduced with permission.^68^ Copyright 2020, World Scientific Publishing Company. (D) SEM image of AuNPs generated by the microfluidic chip combined with a laser‐induced‐graphene heater. Reproduced under terms of the CC‐BY license.^69^ Copyright 2021, The Authors, published by Springer Nature. (E) Diagram and image of a 3D printed microfluidic device for metal NPs synthesis. Reproduced with permission.^70^ Copyright 2019, Elsevier. (F) Schematic diagram of different microfluidic channels for the synthesis of branching AuNPs. Reproduced under terms of the CC‐BY license.^71^ Copyright 2022, The Authors, published by the Royal Society of Chemistry.

Gold and silver NPs (AgNPs) are among the most widely studied metal NPs because of their high stability, ease of synthesis, and good optical properties.^62^ ^c^ Recently, the synthesis of gold and silver nanomaterials has been optimized using microfluidics to control the NPs' size and structure and improve their function as well as production efficiency. Verma and colleagues developed a microfluidic chip with a continuous circular serpentine by which the NPs' nucleation and growth stages could be separated (Figure 5A(i)).^65^ Ye et al. designed S‐shaped microfluidic channels for seed‐mediated synthesis of gold nanobipyramids (Figure 5A(ii)).^66^ In addition, researchers are constantly innovating in chip design to better enhance the mixing of reagents. For instance, Kwak et al. designed a modular microfluidic chip containing a static mixer in addition to two T‐junctions to effectively synthesize AgNPs with controllable size under optimized parameters (Figure 5B).^67^ Liu et al. designed a bilayer Y‐layer microfluidic chip that could make fluids split and recombine, resulting in the controllable synthesis of AgNPs was achieved due to effective mixing (Figure 5C).^68^ Considering the requirements for a controllable, uniform, and stable temperature environment, Srikanth et al. designed a laser‐induced‐graphene heater and integrated it with droplet microfluidics to synthesize AuNPs with good electrochemical properties (Figure 5D).^69^

It is worth mentioning that new technologies, such as 3D printing have been applied to the construction of microfluidic chips that meet the reaction conditions for metal NPs synthesis. For example, Bressan et al. applied a fused deposition modeling (FDM)‐based 3D‐printed microfluidic device to the synthesis of AuNPs and AgNPs. A segmented flow was created by introducing an oil flow perpendicular to one of the reactant flow to avoid the precipitation problem (Figure 5E).^70^ In addition, in recent years, the microfluidic synthesis of metal NPs has also benefitted from the integration of computational modeling, which helps predict the reaction behavior, the effect of the parameters, as well as the properties of the final product.^72^ Dawson and colleagues explored computational modeling to prepare AuNPs with branching shapes (Figure 5F).^71^ The computational analysis method allowed for the identification of key factors predicting the reaction process and the final shape.

**FIGURE 6:**
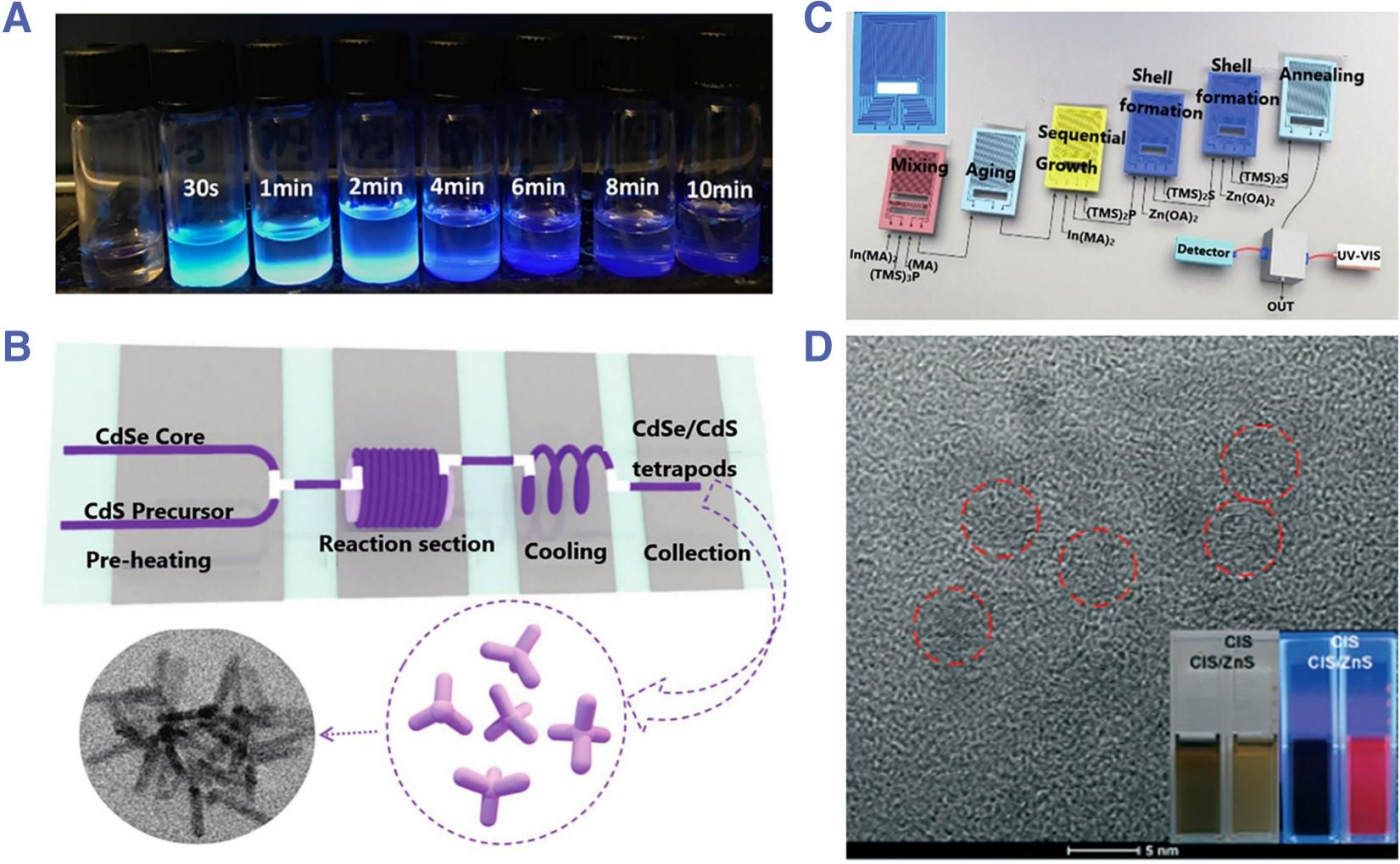
(A) Image of different ZnSe QDs in toluene with increasing retention time (from left to right). Reproduced with permission.^75^ Copyright 2018, American Chemical Society. (B) Schematic diagram of the microfluidic reactor for the synthesis of tetrapod CdSe/CdS QDs. Reproduced with permission.^76^ Copyright 2021, The Royal Society of Chemistry. (C) Multi‐stage microfluidic reactor platform for the generation of InP/ZnS core/shell QDs. Reproduced with permission.^77^ Copyright 2018, John Wiley and Sons. (D) TEM picture of CuInS_2_/ZnS QDs and digital photographs of CuInS_2_ and CuInS_2_/ZnS QDs without and with UV irradiation. Reproduced with permission.^78^ Copyright 2020, The Royal Society of Chemistry.

QDs have a wide range of applications in optical sensing, and their optical properties are closely correlated with their size. Microfluidic synthesis of QDs has been extensively explored since it can achieve great control of the operation parameters and increase the reproducibility between batches.^73^ Microfluidic synthesis of QDs has attracted increasing interest and can be implemented in either continuous flow systems or droplet‐based systems, and the preparation of heterogeneous QDs or core‐shell structured QDs has been demonstrated.^74^ In recent years, some new discoveries have been made on microfluidic synthesis of QDs. For example, Guidelli et al. produced low‐toxicity and radioluminescent ZnSe nanocrystals as substitutes for conventional Cd‐ and Pb‐based QDs (Figure 6A).^75^ Xu and colleagues proposed a low‐temperature synthesis strategy of CdSe/CdS QDs in tetrapod, creating a green and economic approach (Figure 6B).^76^ Baek et al. proposed a high‐temperature and high‐pressure chip platform to fabricate different types of core‐shell QDs (Figure 6C). The reactor had different heating profiles as well as flow distributions, enabling precise control over the reaction processes.^77^ Hu et al. invented a one‐step automated method to synthesize denatured bovine serum albumin (dBSA)‐functionalized CuInS2/ZnS QDs (Figure 6D). The resultant biofunctionalized QDs exhibited a long fluorescence lifetime and could be further modified to perform bioimaging studies.^78^

In addition to the aforementioned systems, microfluidics has also been implemented for the synthesis of other optical nanomaterials, for example, perovskite NPs, organic dyes, UCNPs, etc.^79^ Overall, the capabilities of the microfluidic technology, including the accurate control of reagent flow rate, reaction temperature, residence time, etc., have greatly prompted the development of optical nanomaterials with finely controlled size, morphology, and optical properties favorable for sensing applications.

### 4.2 Fluidic‐templated synthesis of optical materials

Based on several fluid types (continuous flow, segmented flow, and droplet‐based flow), microfluidics can not only accurately synthesize optical nanomaterials^9^ ^a^ but also utilize different fluid configurations as templates to construct microscale optical materials, such as microspheres, microcapsules, microfibers, etc.^80^ The optical properties of these materials can come from the intrinsic optical features of the nanomaterials being incorporated, or being originated from assembly of nanoscale building blocks. Besides, the flexible shape control of these materials as well as the integration of other functional components further enrich the applications of optical materials in sensing and many other fields.

**FIGURE 7:**
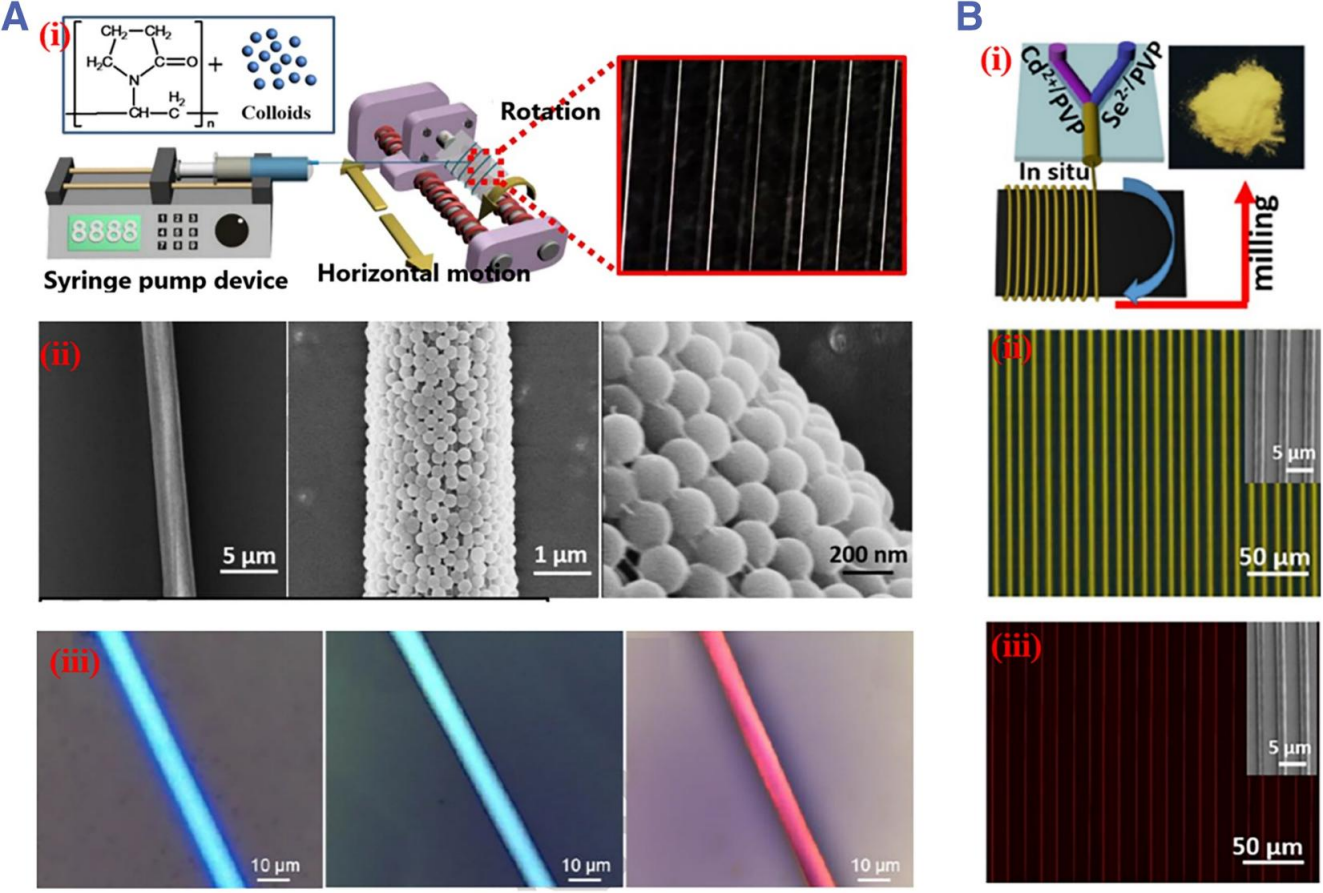
(A) (i) Schematic diagram of the microfluidic‐spinning process; (ii) SEM images of (PVP)/colloidal PhC fibers (CPCFs) under different magnifications; (iii) microscope images of blue, green, and red CPCFs. Reproduced with permission.^82^ Copyright 2019, Elsevier. (B) (i) Schematic diagram of in situ fabrication of CdSe QDs by Y‐type microfluidic platform; (ii, iii) fluorescence microscopy images of (ii) yellow and (iii) red fibers. Reproduced with permission.^83^ Copyright 2018, American Chemical Society.

Microfibers can be continuously generated through microfluidic spinning. The size, structure, morphology, and surface characteristics of microfibers can be well regulated by tuning the microfluidic channel geometry, fluid rheology, and flow parameters.^81^ To endow microfibers with specific optical properties, Chen and colleagues prepared colloidal PhC microfibers based on a microfluidic rotation technique. An ethanol suspension of silica colloids containing polyvinylpyrrolidone (PVP) formed the precursor that was spun and drawn to form fibers. After ethanol volatilization and the removal of PVP through calcination, the silica colloids assembled to form colloidal crystal microfibers with structural colors (Figure 7A). Factors such as the size of the colloids and the rotational speed were important factors affecting the product properties. Polyacrylamide (PAM) was used to give rise to humidity‐sensing ability.^82^ In addition to inducing fiber‐templated colloidal assembly, the same group developed a microfluidic system integrating fiber spinning with in situ synthesis of QDs. A Y‐shaped microfluidic chip was constructed to introduce two precursor solutions containing Cd^2+^ and Se^2−^, respectively. The resultant fibers were annealed to promote solvent evaporation during QD generation. By adjusting the precursor solution concentrations, various fluorescent fibers were obtained (Figure 7B).^83^

**FIGURE 8:**
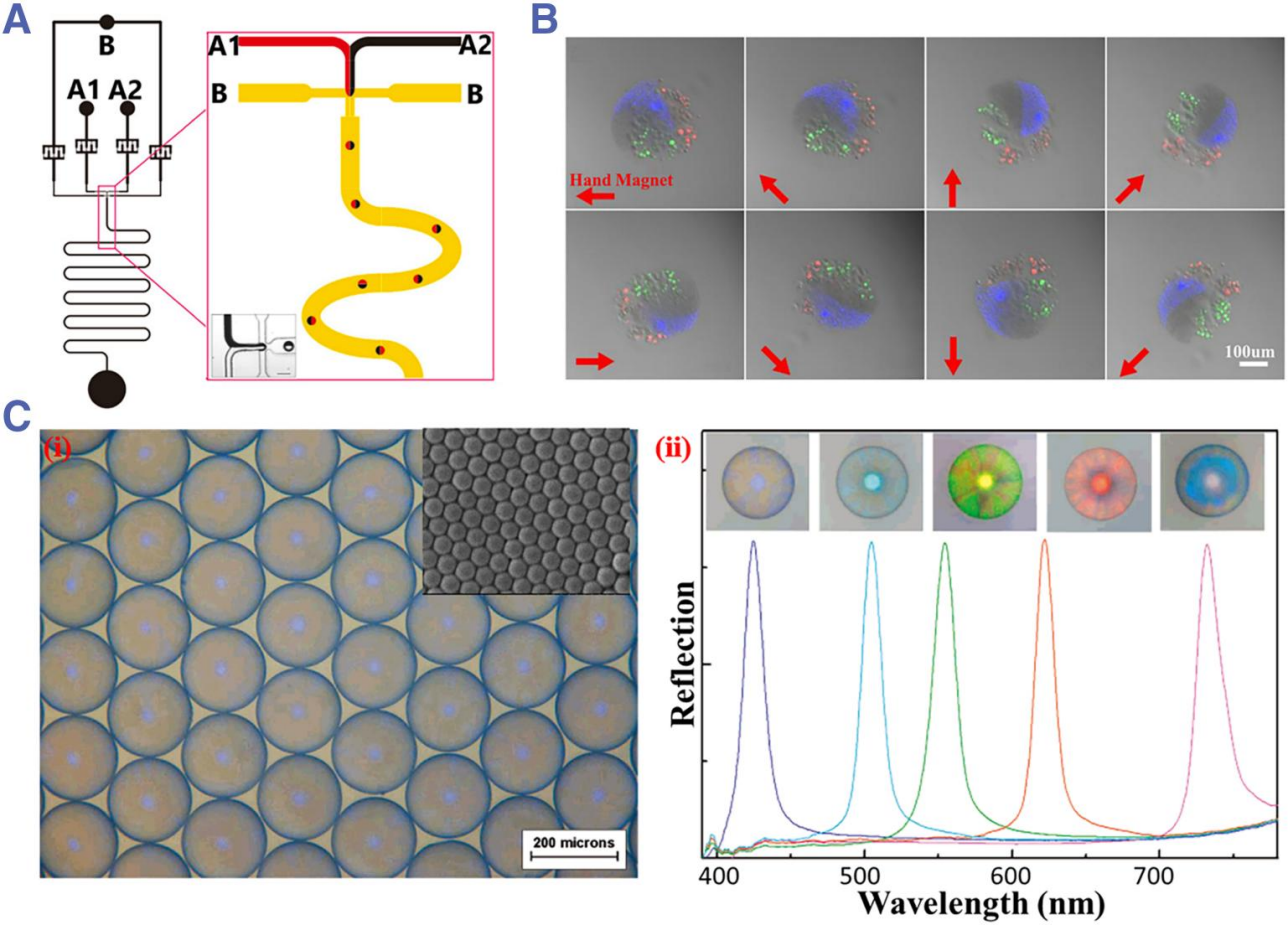
(A) Schematic illustration of the fabrication of fluorescent magnetic multifunctional Janus microspheres. Reproduced with permission.^86^ Copyright 2016, Elsevier. (B) Rotational movement of multi‐compartmental microspheres under the action of magnet. Reproduced with permission.^87^ Copyright 2019, John Wiley and Sons. (C) (i) Microscopic and SEM images of silica colloidal crystal; (ii) reflective spectra of colloidal crystal microspheres composed different sizes of silica NPs. Reproduced with permission.^88^ Copyright 2008, American Chemical Society.

Droplet microfluidics offers a facile way of fabricating solid microspheres through various curing processes such as photopolymerization, ionic‐crosslinking, thermal curing, etc.^8^, ^84^ In addition to simple microspheres, Janus and multicompartment microspheres with different components can also be obtained by simultaneously emulsifying parallel biphasic/multiphase fluids at the same time.^85^ These features have been exploited for the preparation of microspheres with fascinating optical properties. He's team prepared fluorescent magnetic multifunctional Janus microspheres using droplet microfluidics. The experiment setup involved the use of two alginate fluids containing Fe_3_O_4_ NPs and CdSe/ZnS QDs, respectively, which flowed together into a flow‐focusing channel and pinched off to form Janus droplets. The droplets then underwent ionic cross‐linking reactions with Ca^2+^ in a serpentine channel to form Janus microspheres with excellent magnetic/fluorescent properties (Figure 8A).^86^ In addition to Janus microspheres, Wu et al. designed a flow‐focusing microfluidic platform consisting of a horizontal upstream chip and a vertical downstream device. Multiple streams were converged and simultaneously emulsified to form droplets, which were then converted into multi‐compartmental microspheres (Figure 8B). Fluorescent NPs, magnetic beads, and different types of cells could be loaded within separate compartments, thus exerting multiple functions.^87^ Besides, colloidal crystal microspheres can be generated by the confined assembly of colloidal NPs in droplets. Zhao's team designed a microfluidic device with a T‐shaped channel for the preparation of size‐controlled, monodisperse silica colloidal crystal microspheres (Figure 8C). The resultant microspheres possessed a closely packed microstructure and tunable structural colors, allowing them to be used in a broad range of sensing applications.^88^ In addition to spherical colloidal crystal microparticles, the same group further proposed a phase separation strategy to fabricate anisotropic colloidal crystal microparticles, which served as a cell monitoring platform.^89^

**FIGURE 9:**
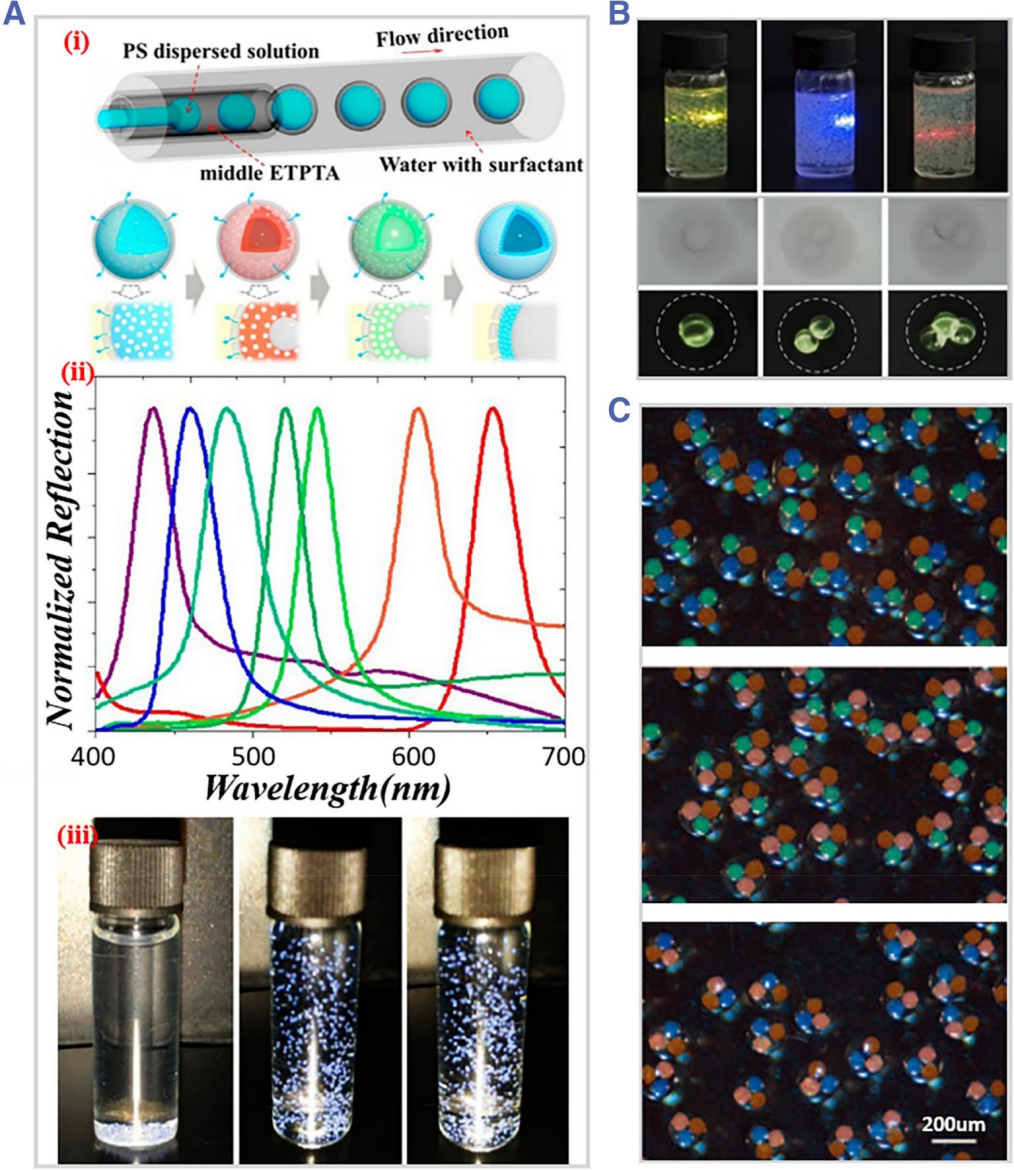
(A) (i) Schematic diagram of the preparation of the hollow PhC microcapsules; (ii) the reflection spectra of different PhC bubbles; (iii) presenting of the PhC bubbles' suspending property. Reproduced with permission.^53^ Copyright 2015, American Chemical Society. (B) Photograph and microscopic images of different UCNPs‐encapsulated microcapsules under NIR excitation. Reproduced with permission.^93^ Copyright 2022, Elsevier. (C) Microscopic images of the multi‐core structural color microcapsules. Reproduced with permission.^94^ Copyright 2012, The Authors, published by Springer Nature.

Microcapsules typically have a solid shell and a liquid, solid, or gas core.^90^ The distinctive core‐shell structure and flexibility in material choice endow microcapsules with diverse functions.^91^ Microcapsules can be derived from double or higher‐order emulsion droplets by curing the shell or via phase separation, interfacial reaction, and assembly in single emulsion droplets.^92^ Besides, microcapsules with tailored structures and morphologies, for example, single‐core, multi‐core, multi‐layer, etc., could be obtained,^90^ which further expand their application values. Microcapsules with optical properties can also be derived from confined colloidal assembly or direct encapsulation of optical nanomaterials. Shang et al. prepared hollow photonic microcapsules with a polymer shell, a PhC inner layer, and a gaseous core through cavitation‐induced colloidal assembly. The PhC inner layer gave rise to vivid structural colors and the gaseous core made the hollow microcapsules suspended in a solution (Figure 9A).^53^ Wang et al. fabricated hydrogel microcapsules containing UCNPs cores through electrospray microfluidics. The anti‐Stokes luminescent property of the encapsulated UCNPs made the microcapsules emit visible light under near infrared (NIR) stimulation (Figure 9B).^93^ In addition, microcapsules containing cores of different structural colors were achieved by co‐encapsulating multiple non‐closely‐packed colloidal crystal arrays (Figure 9C).^94^ The number of cores and the color combinations were determined by the system parameters, such as the channel configuration, the flow rate, etc. The multicore nature could avoid the interference of the color of each core, making the microcapsules promising optical materials for sensing applications.

## 5 APPLICATIONS OF MICROFLUIDIC‐DERIVED OPTICAL SENSORY MATERIALS

As mentioned above, optical sensors offer tremendous advantages since they allow direct, real‐time, and, in specific cases, label‐free measurements of many biological and biochemical signals with the advantages of high specificity, high sensitivity, and low cost. In the last decades, the materials research and technological development of optical sensors have shown exponential growth, allowing them to find extensive applications in such biomedical fields as disease diagnosis, drug evaluation, and organ‐on‐a‐chip.

### 5.1 Disease diagnosis

**FIGURE 10:**
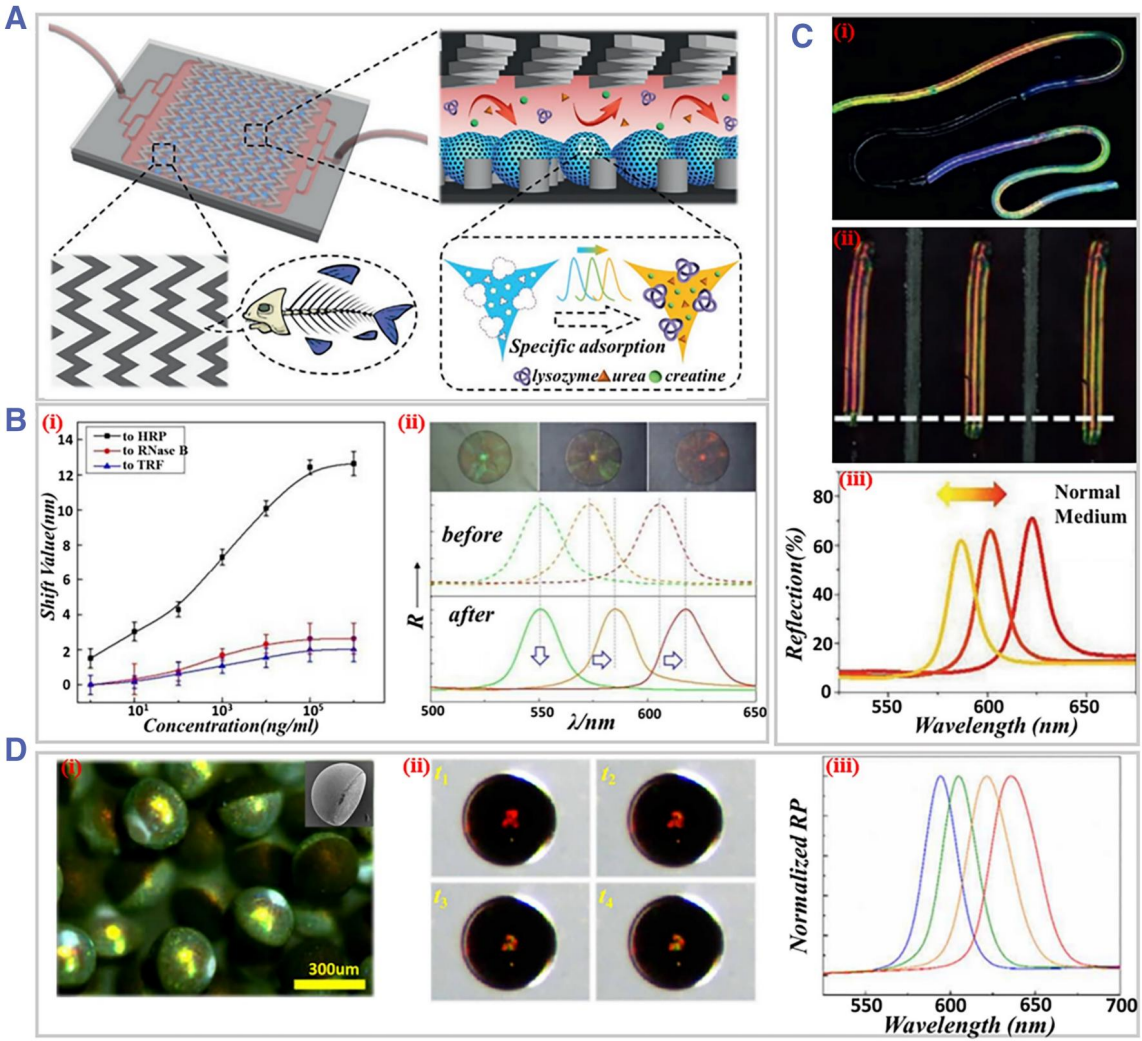
(A) Schematic illustration of the inverse opal particle‐integrated herringbone microfluidic platform for detection of urea, creatine, and lysozyme. Reproduced with permission.^99^ Copyright 2020, John Wiley and Sons. (B) (i) Plot of the response of horseradish peroxidase (HRP) blotting of inverse opal particles against different concentrations of HRP, RNase B, and TRF; (ii) microscopic images and reflection spectra of different colors of PhC barcodes blotted with TRF, RNase B, and HRP, respectively, prior to and after immersed in a solution only with HRP and RNase B. Reproduced with permission.^100^ Copyright 2016, The Royal Society of Chemistry. (C) (i) Optical image of heterogeneous structural color microfibers; (ii) optical microscope image of the structural color change of the microfiber in response to cardiac contraction; (iii) the corresponding shift of the reflection peak wavelength. Reproduced with permission.^101^ Copyright 2021, John Wiley and Sons. (D) (i) Microscopic and SEM images of Janus structural color particles; (ii) images of the structural color‐changing process of a hydrogel Janus SCP in response to myocardial contraction; (iii) the corresponding reflection spectra (right to left). Reproduced with permission.^89^ Copyright 2020, The Authors, published by American Association for the Advancement of Science.

Molecular biomarkers, for example, proteins, nucleic acids, and small molecules in saliva, blood, urine, and other body fluids are helpful for the identification of disease status.^95^ To this end, optical sensors provide a convenient way for converting biological signals into detectable optical signals. Besides, optical sensors could be integrated into a miniature platform, providing fast response times, low reagent consumption, ease of operation, and the ability to perform multiplex assays under specific circumstances. These features render optical sensors a high position in the diagnosis of cancer, infectious diseases, metabolic diseases, etc.^96^ Remarkably, microfluidic‐derived optical sensor materials own flexible and controllable structural and optical features and thus have brought new platforms for disease diagnostics^97^. Zhao's team proposed a series of colloidal PhC microspheres as sensing platforms to quantify various biomarkers.^98^ For example, a novel blood purification chip was constructed by integrating porous inverse opal microparticles in a herringbone mixer. The porous structure and optical sensing ability of the inverse opals, along with the use of the herringbone mixer, enabled visualized detection of multiple molecules including urea, creatine, and lysozyme (Figure 10A).^99^ It is worth noting that the structural color of the colloidal crystal microspheres can serve as distinguishable barcodes to realize multiplexed bioassays. With this, Zhao and colleagues proposed a PhC barcode‐based suspension array technique for multiplex label‐free analysis of glycoprotein. The barcode particles were composed of borate affinity molecularly imprinted polymer (MIP) and possessed an inverse opal structure. Binding of glycoprotein resulted in the swelling of MIP and thus a color shift of the barcodes. Due to the high specificity of the binding reaction, this platform could be utilized for multiplex quantitative analysis of glycoproteins (Figure 10B).^100^

In addition, microfluidic‐derived optical sensor materials can exhibit not only excellent optical properties but also biocompatibility, making them capable for cell sensing. This also endow them with potential values in disease diagnostics. Chen et al. prepared a type of multi‐colored heterogeneous structured color microfiber for cellular mechanics sensing by microfluidic spinning. The microfiber comprised non‐close‐packed colloid crystal arrays segments for optical sensing and methacrylated gelatin (GelMA) hydrogel segments for cell culture. As such, the beating of the cultivated cardiomyocytes resulted in the stretch of the structural color section of the fibers, thus converting cell mechanics signal into visualized optical information (Figure 10C).^101^ Alternatively, Wang et al. presented GelMA anisotropic Janus structural color particles for three‐dimensional (3D) cardiomyocytes culture and monitoring. The bright structural color of the particles and their dynamic response to cardiomyocytes' beating frequency and strength made it an important platform for evaluation of cardiomyocytes status (Figure 10D).^89^ In addition, the fluorescence‐based sensors, including those encoded microcarriers, have a broad application in multiplex assays and also play an important role in multi‐molecule detection and disease diagnosis.^102^

### 5.2 Drug evaluation

**FIGURE 11:**
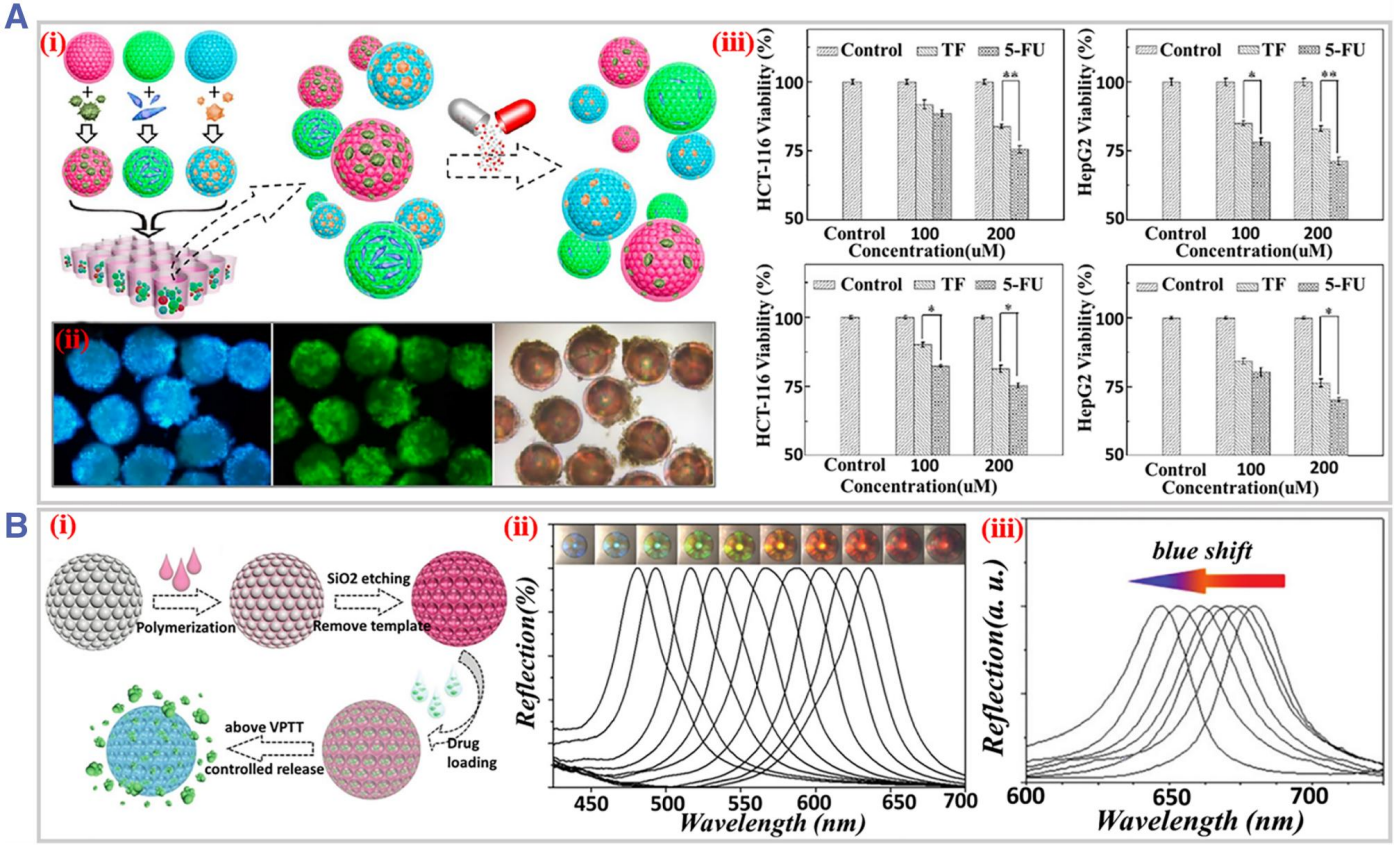
(A) (i) Schematic illustration of the cell PhC co‐cultural platform for drug screening; (ii) images of cell attachment on core‐shell PhC particles; (iii) MTT assay results. Reproduced with permission.^54^ Copyright 2016, American Chemical Society. (B) (i) Schematic diagram of the preparation of the inverse opal particles and the temperature‐controlled drug release process; (ii) optical microscope images and the corresponding reflection spectra of the inverse opal particle at varying temperatures; (iii) the blue shifted reflection spectra corresponding to different cycles of temperature‐triggered release. Reproduced with permission.^104^ Copyright 2015, The Royal Society of Chemistry.

The development of drug evaluation platforms is urgently needed, and microfluidic‐derived optical sensing materials brings new opportunities to this field. The structural features and chemical compositions of these materials can be well controlled to enable 3D cell culture, simulate microenvironment in vivo, and monitor drugs’ effects on the human body. Additionally, their excellent optical information enables them to get the information about drug screening easily without harming the cellular state. Thus, microfluidic‐derived optical sensing materials are emerging as important tools for the research of drug development.^103^ Fu et al. proposed core‐shell PhC microspheres for drug evaluation. The shell of the microspheres, GelMA can simulate the 3D ECM microenvironment and promote cell adhesion and growth. The core of the microspheres exhibited a PhC structure that provided stable optical encoding to monitor biochemical processes and identify the responses of different cell types. In a proof‐of‐concept study, hepatocellular carcinoma cells (HepG2), colon cancer cells (HCT‐116), and normal fibroblast cells NIH‐3T3 were cultured on microspheres of different colors and formed spheroids. Cytotoxicity test of the drug tegafur (TF) was then performed, which is a prodrug of 5‐fluorouracil (5‐FU) that takes effect only after liver enzymatic reaction. The test results showed that the toxicity of TF was significantly enhanced in the presence of HepG2 spheroids‐loaded microspheres, indicating that some of the liver function was reproduced (Figure 11A).^54^

The release and delivery of drug is an important aspect, and microfluidic‐derived drug carriers have been widely studied. Notably, if these carriers own optical sensing capabilities, the drug release process can be monitored in real time. Zhang et al. developed inverse opal particles based on poly(N‐isopropylacrylamide) (pNIPAM) hydrogel for controlled drug release and self‐monitoring of it. Due to the temperature sensitivity of pNIPAM, the release of loaded drugs can be regulated through the contraction and expansion of the particles via temperature control. More intriguingly, during the release process, the optical information of the inverse opal, that is, the reflection peak blue shifted, thus facilitating real‐time monitoring of drug release (Figure 11B).^104^ Besides, the SERS‐based sensor has low interference under aqueous solvents, which is a highly sensitive analytical tool for quantitative detection of low‐dose drugs in liquid and solid samples, and has great values in therapeutic drug monitoring.^22^ ^b,^ ^105^

### 5.3 Organ‐on‐a‐chip

Organ‐on‐a‐chip is a cell culture system that reproduces the structure and function of human organs, effectively mimics the in vivo cell growth environment, and monitors the conditions and stimuli‐responses of cells.^106^ These features make it a powerful platform in biomedical fields and a hopeful replacement for traditionally used animal models.^107^ Sensor materials can be integrated in an organ‐on‐a‐chip system for harmless and real‐time monitoring of various parameters, and particularly, optical sensors can offer a convenient way for this purpose. For example, heart‐on‐a‐chip systems have been constructed with PhC films integrated as key sensor units for monitoring the status of cardiomyocytes and testing their response to drugs.^108^ As for microfluidic‐derived optical sensor materials, the aforementioned structural color microfibers capable for cell mechanics sensing has been incorporated in organ‐on‐a‐chip systems. Benefitting from the microscale size of the fibers, single‐cell‐level sensing was achieved.^54^, ^89^, ^101^ Similarly, the aforementioned structural color particles possess more flexible shape and morphology compared to films or fibers, which can be expected in future organ‐on‐a‐chip systems. Apart from structural color materials utilized for cell mechanics sensing, other types of microfluidic‐derived optical sensor materials can be integrated in organs‐on‐chips systems for converting various biological signals into detectable optical signals and providing feedback information.

## 6 CONCLUSION AND OUTLOOK

Optical sensors transform sample signals into detectable optical signals, which makes them increasingly important in biomedical, pharmaceutical, and healthcare applications. In recent years, the generation and optimization of optical sensor materials with the use of microfluidic technology have promoted the application of optical sensors in various fields. Here, we focused on the up‐to‐date progress of microfluidic‐derived optical sensor materials and their applications in the biomedical field. We introduced the mechanisms of several commonly used optical sensing methods and the corresponding materials with sensing capacities. Then, we discussed the role of microfluidics in the generation of these materials and the control of their structural and functional features. Finally, we list some of the applications of microfluidic‐derived optical sensor materials in the biomedical field. With that, we hope to emphasize the new design paradigms of optical sensors with the help of microfluidics and inspire future research on both fundamental and technical aspects.

Despite significant progress of microfluidic‐derived optical sensor materials, challenges remain in designing and pioneering new materials and expanding their biomedical applications. Firstly, the optical and other properties of sensor materials need to be further improved, especially when considering biomedical applications. This includes the stability of some NPs, the affinity to biological analytes, the biocompatibility, etc.^109^ In addition, the role of microfluidic‐derived optical sensor materials for sensing at the cell level and body level is relatively lacking, and this area could be a focus of future research. In particular, the role of microfluidic‐derived optical sensor materials in organ‐on‐a‐chip or organoid‐on‐a‐chip systems has not yet been fully exploited. We expect that, with the development of multi‐organ‐on‐a‐chip, multi‐parameter sensors can be combined, together with other functional units (e.g., sample loading, processing, and rection). Thus, a highly integrated in vitro system could be constructed for simulating multiorgan interactions and converting this information into optical signals with accuracy. In addition, currently available optical sensors are mainly in vitro systems, yet the construction of in vivo human body optical sensors is a future research direction. To this end, the long‐term biocompatibility, biosafety, and effect of body parameters on sensor sensitivity are major challenges to be addressed. Finally, the improvement of signal processing methods and the need for creating portable, low‐cost, and easy‐to‐operate optical sensing devices would foster the innovation of optical sensor materials, which, in turn, pushes forward the microfluidic technique. Overall, although challenges remain, we foresee continuous achievements in this area with the joint efforts in optics, material science, biomedicine, and related technologies.

## AUTHOR CONTRIBUTION

Qiao Wang wrote the manuscript. Luoran Shang revised the manuscript. Chong Wang, Xinyuan Yang, Jiali Wang and Zhuohao Zhang contributed to scientific discussion.

## CONFLICT OF INTEREST STATEMENT

The authors declare that they have no conflict of interest. Luoran Shang is a member of the *Smart Medicine* editorial board.
